# Population Pharmacokinetics of Linezolid in Elderly Hospitalized Patients: Implications for Therapeutic Drug Monitoring

**DOI:** 10.3390/pharmaceutics18050528

**Published:** 2026-04-27

**Authors:** Gloria Gallego-Hernández, Andrea Albarrán-Gómez, José Germán Sánchez-Hernández, Jaime Cándido García-Casanueva, María José Otero

**Affiliations:** 1 Pharmacy Service, University Hospital of Salamanca, 37007 Salamanca, Spain; 2 Biomedical Research Institute of Salamanca (IBSAL), 37007 Salamanca, Spain; 3 Department of Pharmaceutical Sciences, Faculty of Pharmacy, University of Salamanca, 37007 Salamanca, Spain

**Keywords:** linezolid, therapeutic drug monitoring, elderly patients, model-informed precision dosing

## Abstract

**Background:** Linezolid is widely used for the empirical and targeted treatment of Gram-positive infections. Elderly patients frequently exhibit substantial pharmacokinetic variability due to age-related physiological changes and high comorbidity burden, which may predispose to drug accumulation and toxicity. This study aimed to develop and evaluate a population pharmacokinetic (PopPK) model of intravenous (IV) linezolid in elderly patients (65–87 years) to support therapeutic drug monitoring and explore exposure-risk scenarios associated with overexposure. **Methods:** A retrospective single-center study including 149 patients and 293 serum trough concentrations was conducted. Patients were randomly assigned to development (n = 103) and independent validation (n = 46) cohorts. Linezolid concentrations were quantified using an enzyme immunoassay. The PopPK model was developed in NONMEM^®^ using FOCE-I. Model performance was evaluated using standard diagnostic plots, bootstrap analysis, visual predictive checks, and validation metrics (mean prediction error [MPE] and mean absolute prediction error [MAPE]). Monte Carlo simulations assessed the probability of overexposure (Cmin > 8 mg/L) and the probability of target attainment (PTA; AUC24/MIC ≥ 100) under standard dosing (600 mg IV every 12 h). **Results:** Linezolid pharmacokinetics were best described by a one-compartment model with first-order elimination. Estimated glomerular filtration rate, treatment duration, and age were identified as significant predictors of clearance. Internal and independent validation confirmed the robustness and predictive performance of the model. Simulations showed a high probability of overexposure in patients with impaired renal function, particularly during prolonged treatment. **Conclusions:** Renal function, age, and treatment duration are major determinants of linezolid exposure in elderly patients. Standard dosing frequently results in overexposure, supporting early therapeutic drug monitoring and individualized dose adjustment in this vulnerable population.

## 1. Introduction

Linezolid is a synthetic oxazolidinone antibiotic that inhibits bacterial protein synthesis and is widely used for the treatment of Gram-positive infections, including multidrug-resistant pathogens such as methicillin-resistant *Staphylococcus aureus* (MRSA) and vancomycin-resistant *Enterococcus faecium* (VRE). Beyond its approved indications for pneumonia and complicated skin and soft tissue infections, linezolid is frequently prescribed in hospitalized patients as both targeted therapy and empirical Gram-positive coverage [1,2,3].

According to the Summary of Product Characteristics, the recommended intravenous (IV) dose is 600 mg every 12 h; however, growing evidence indicates that this fixed regimen may not be optimal for all patients [3,4]. Therapeutic drug monitoring (TDM) has therefore been proposed to optimize exposure, targeting an area under the concentration–time curve to a minimum inhibitory concentration ratio (AUC/MIC) of >80–100 for efficacy and trough concentrations (Cmin) between 2 and 7 mg/L to minimize toxicity while maintaining antibacterial activity [1,2,5].

Although dose adjustment is not routinely recommended in elderly patients, several real-world studies have demonstrated a significant association between advanced age and increased linezolid exposure [6,7,8,9]. In a large observational cohort, Cattaneo et al. reported supratherapeutic trough concentrations in approximately 30% of patients younger than 65 years, 50% of those aged 65–80 years, and 65% of patients older than 80 years, indicating a clear age-dependent risk of overexposure [10].

Advancing age is associated with progressive physiological changes affecting organ function, body composition, and drug elimination pathways, which may significantly influence linezolid pharmacokinetics and promote drug accumulation. Linezolid undergoes predominantly non-enzymatic oxidative metabolism. Approximately 30% of the administered dose is excreted unchanged in urine, and nearly 50% is recovered as inactive metabolites that are mainly renally cleared. Therefore, renal function is an important determinant of linezolid exposure. In a large observational study, Fresán et al. identified renal dysfunction, liver cirrhosis, and higher dose-to-body weight ratios as major predictors of supratherapeutic linezolid concentrations [11]. Renal impairment, in particular, has been associated with up to 1.6-fold higher levels compared with patients with preserved renal function [12].

Furthermore, polypharmacy is common in elderly patients and may contribute to variability in linezolid exposure through drug–drug interactions. Despite its predominantly non-enzymatic metabolism, interactions (e.g., involving transport proteins such as P-glycoprotein) may still influence its pharmacokinetics, supporting the need for TDM [13,14].

Collectively, these age-related pharmacokinetic alterations have clinically significant consequences. Across multiple studies, elevated linezolid Cmin (>7–8 mg/L) has been consistently associated with an increased risk of hematological toxicity, particularly thrombocytopenia and anemia [5,8,10,15]. In a large prospective cohort, hematological adverse events were the most frequent, with thrombocytopenia being independently associated with Cmin >8 mg/L and prolonged treatment duration, while renal impairment, advanced age, and high comorbidity burden were identified as major risk factors [16].

Considering the narrow therapeutic index of linezolid and the increased risk of overexposure and treatment-related toxicity in elderly patients, robust dose optimization strategies are needed. Population pharmacokinetic (PopPK) modeling provides a powerful framework to identify clinically meaningful covariates and support individualized dosing via Bayesian forecasting. However, data specifically focused on elderly hospitalized patients remain limited, particularly in cohorts reflecting routine TDM practice [2].

The present study was designed to develop and evaluate a PopPK model of linezolid in elderly patients (65–87 years) to support TDM and safety-oriented Bayesian dose individualization. A secondary objective was to explore simulation-based exposure scenarios in order to identify patient subgroups at increased risk of overexposure and to support early TDM strategies in this particularly vulnerable population.

## 2. Materials and Methods

### 2.1. Study Design and Population

This was a retrospective, single-center, multidisciplinary study conducted at a tertiary-care hospital. The study was approved by the Ethics Committee of Hospital Universitario de Salamanca (protocol code PI 2026 01 2177, approved on 24 January 2026), which waived the requirement for informed consent due to the retrospective nature of the study.

Adult patients aged 65–87 years receiving IV linezolid for either targeted or empirical therapy of Gram-positive bacterial infections between January 2024 and September 2025 were included. IV linezolid was prescribed as part of routine clinical care according to the treating physician’s clinical judgment, microbiological data when available, and local prescribing practice, particularly when resistant Gram-positive pathogens were suspected or when alternative therapies were not considered appropriate. Exclusion criteria were treatment with oral linezolid, active oncological or hematological disease, renal replacement therapy, and critical illness requiring ICU admission. All consecutive patients meeting the predefined inclusion and exclusion criteria during the study period were retrospectively identified from routine TDM records. Patients with incomplete data or without available linezolid concentration measurements were also excluded. Patients were randomly assigned in an approximately 2:1 ratio to the PopPK model development dataset and an internal independent validation cohort.

All patients initiated treatment with linezolid 600 mg every 12 h, administered as a 1-h infusion. Thereafter, dosing was individualized based on the TDM results. According to the local TDM protocol, the first serum linezolid concentration (SLC) was obtained immediately before the third to fifth dose. Subsequent samples were generally collected every 3–4 days during continued therapy, and always immediately before the next scheduled dose in order to measure Cmin. The exact sampling time relative to the last dose was recorded for each sample and incorporated into the pharmacokinetic analysis. Thus, “time since last dose” reflected the actual recorded interval between the last administration and sampling, rather than the nominal 12-h dosing interval. The TDM target was a Cmin between 2 and 7 mg/L. Concentrations > 8 mg/L were avoided to minimize toxicity and treatment discontinuation.

The following patient characteristics were collected:

- Demographic variables: age, sex, body weight, height, ideal body weight (IBW), adjusted body weight (using a 0.4 correction factor for excess body weight), body mass index (BMI: weight/height^2^), and body surface area (BSA: calculated using the Mosteller formula).
- Treatment variables: dosing regimen, total number of administered doses, and overall treatment duration. Information on concomitant medications with potential pharmacokinetic interaction was also recorded, with particular attention to known enzyme or transporter inducers and inhibitors, including rifampicin and macrolides.
- Laboratory parameters: serum albumin, total protein, lactate dehydrogenase (LDH), alanine aminotransferase (ALT), aspartate aminotransferase (AST), total bilirubin (BLT), serum creatinine, C-reactive protein (CRP), procalcitonin, hemoglobin, and platelet count. Renal function was estimated using the Cockcroft–Gault (CG) and CKD-EPI equations. For modeling purposes, the CKD-EPI estimated glomerular filtration rate (eGFR) expressed in absolute values (mL/min) was used. For modeling purposes, eGFR values were capped at 130 mL/min to avoid implausible estimates and to limit undue leverage of extreme values on the eGFR–CL relationship. This was considered particularly relevant in this elderly population, in whom reduced muscle mass may lead to artificially low serum creatinine and overestimation of renal function. Renal function was handled as a time-varying covariate, using the value closest to each concentration measurement.

### 2.2. Determination of Linezolid Concentrations

SLC was measured in the Clinical Pharmacokinetics Laboratory of the Pharmacy Department using an enzyme immunoassay (ARK™ Linezolid Assay, ARK Diagnostics, Fremont, CA, USA) on an Abbott Architect ci4100 automated analyzer (Abbott Diagnostics, Abbott Park, IL, USA). Serum samples were obtained from whole blood collected in tubes without anticoagulant, centrifuged at 3000 rpm for 10 min. Samples were analyzed immediately after processing, without long-term storage prior to quantification.

The lower limit of quantification (LLOQ) of the assay was 0.8 mg/L, with a calibration range from 0.8 to 30 mg/L. According to the manufacturer’s method-comparison study, the assay showed good agreement with LC-MS/MS, with limited reported cross-reactivity for the main inactive linezolid metabolites. SLC values below the LLOQ were excluded from the formal analysis [17].

### 2.3. Population Pharmacokinetic Model Development

Population pharmacokinetic analysis was performed using nonlinear mixed-effects modeling with the First-Order Conditional Estimation method with interaction (FOCE-I) implemented in NONMEM^®^ version 7.5 (ICON Development Solutions, Ellicott City, MD, USA) [18,19]. Data processing, graphical diagnostics, and simulation-based analyses were conducted in R (R Foundation for Statistical Computing, Vienna, Austria) [20]. Stepwise covariate modeling procedures were supported using Perl-speaks-NONMEM (PsN) version 5.3.1 (Uppsala University, Department of Pharmaceutical Biosciences, Uppsala, Sweden) [21].

Given the IV administration and sampling design, structural models were initially explored using both one- and two-compartment parameterizations with first-order elimination. Although a two-compartment structure and inter-individual variability (IIV) on volume of distribution (V) were evaluated, they did not result in significant improvement in model fit or parameter stability. Therefore, a one-compartment model parameterized in terms of clearance (CL) and V was retained. IIV was assumed to follow a log-normal distribution and was implemented using exponential random-effects models. Residual unexplained variability (RUV) was explored using additive, proportional, and combined error structures. Model discrimination was based on objective function value (OFV), parameter precision, shrinkage assessment, and graphical goodness-of-fit diagnostics (GOF).

Renal function was considered a priori a clinically relevant determinant of linezolid CL and was therefore systematically evaluated during early model development. Multiple estimators of renal function were tested, including absolute eGFR and CG calculated using different body weight descriptors.

Given the observed decrease in CL during the course of treatment, time-dependent effects were formally investigated. Alternative approaches were explored, including a piecewise model and continuous time-varying functions. Nonlinear (capacity-limited) elimination was not pursued further, as the observed concentration range did not support the reliable estimation of nonlinear parameters and the inclusion of time-dependent clearance adequately captured the observed exposure patterns.

Covariate selection beyond renal function and treatment duration was performed using a stepwise covariate modeling strategy implemented through PsN, applying predefined forward inclusion and backward elimination criteria based on changes in OFV. Final model selection was based on statistical robustness, parameter precision, physiological plausibility, and predictive performance [22]. Finite mixture models were not formally explored, as neither the distribution of observed concentrations nor empirical Bayes diagnostics suggested clear latent subpopulations, and the predominantly sparse trough-oriented sampling design was unlikely to support stable and clinically interpretable mixture components.

### 2.4. Model Validation and Predictive Performance

The predictive performance of the final linezolid PopPK model was assessed through a comprehensive evaluation strategy including both internal and independent validation procedures.

Internal model qualification comprised graphical and statistical diagnostics. GOF plots were generated to examine the agreement between the observed serum concentrations and both population-predicted and individual-predicted values. Conditional weighted residuals (CWRES) were evaluated against population predictions and time since last dose to detect potential model misspecification, structural bias, or time-dependent trends [23,24]. Diagnostic plots were visually inspected for symmetry, homoscedasticity, and absence of systematic deviation. Model robustness and parameter precision were further evaluated using a non-parametric bootstrap procedure (1000 replicates) performed with PsN [25]. Bootstrap datasets were generated by random resampling with replacement from the original development cohort. Only successful minimization runs were retained for analysis. Median parameter estimates and their corresponding 95% confidence intervals derived from the bootstrap distributions were compared with the final model estimates to assess stability and reliability. Simulation-based diagnostics were conducted to evaluate the model’s ability to reproduce the observed concentration–time profile and variability structure. Visual predictive checks (VPCs) were generated from 1000 simulated datasets, and observed percentiles were compared with the corresponding simulated prediction intervals across time [26].

Independent validation was conducted using an independent cohort generated through random allocation at the time of study inclusion. The validation cohort was not used during model building or parameter estimation and was reserved exclusively for external predictive performance assessment. Predictive performance in the validation cohort was evaluated using graphical diagnostics and quantitative metrics. Observed serum concentrations were compared with population-predicted and individual-predicted values, and model accuracy and precision were quantified using the mean prediction error (MPE) and mean absolute prediction error (MAPE), respectively. These metrics were calculated to assess systematic bias and overall predictive performance in an independent patient population. Both metrics were interpreted according to internationally accepted criteria for PopPK model validation [24].

$$MPE=\frac{1}{n}\cdot \sum_{i=1}^{n}\frac{C_{pred,i}-C_{obs,i}\;}{C_{obs,i}}\times 100$$

$$MAPE=\frac{1}{n}\cdot \sum_{i=1}^{n}\left|\frac{C_{pred,i}-C_{obs,i}}{C_{obs,i}}\right|\times 100$$

where:

*C_pred,i_*: predicted concentration;

*C_obs,i_*: observed concentration;

*n*: total number of observations.

### 2.5. Simulations and Exposure-Based Target Attainment

Monte Carlo simulations were conducted using the final linezolid PopPK model to characterize exposure patterns across clinically relevant scenarios and to evaluate exposure-based target attainment. Simulation scenarios were defined using representative combinations of demographic, clinical, and laboratory covariates within the range observed in the study population, and concentration–time profiles following multiple dosing were generated until the steady-state was reached. For each scenario, 1000 virtual individuals were generated by sampling pharmacokinetic parameters according to the IIV estimated in the final model. Intravenous linezolid was simulated at the standard dosing regimen of 600 mg every 12 h administered as a 1-h infusion.

The probability of target attainment (PTA) was evaluated according to two exposure metrics: (i) Cmin, using predefined therapeutic thresholds (2–7 mg/L), and (ii) the AUC24/MIC ratio for representative minimum inhibitory concentrations (MIC 1 and 2 mg/L), which were the main pharmacokinetic/pharmacodynamic parameters used to assess exposure in this study. Individual AUC24 values were obtained by the numerical integration of model-predicted concentration–time profiles over each 24-h interval. PTA was calculated as the proportion of simulated individuals achieving the predefined pharmacokinetic targets at clinically relevant time points. Simulation results were summarized graphically using concentration–time profiles and heatmaps illustrating the probability of overexposure and pharmacodynamic target attainment across covariate strata.

## 3. Results

### 3.1. Patient Characteristics

A total of 149 patients contributing 293 quantifiable LSCs were included in the pharmacokinetic analysis. Fifteen additional measurements were below the LLOQ and were therefore excluded from further analysis. Following random allocation, 103 patients (198 concentrations) were assigned to the model development cohort, while 46 patients (95 concentrations) constituted the validation cohort. Baseline demographic and clinical characteristics of the study population are summarized in Table 1. Continuous variables were summarized as the median [interquartile range or range] or mean (standard deviation), as appropriate, and categorical variables as number (percentage). The distribution of observed SLCs over time since treatment initiation is shown in Supplementary Figure S1, further illustrating the predominantly trough-oriented nature of the dataset.

### 3.2. Population Pharmacokinetic Model

Linezolid pharmacokinetics were adequately described by a one-compartment model with first-order elimination, parameterized in terms of CL, and a VA two-compartment model was also evaluated; however, although it produced a modest reduction in OFV compared with the one-compartment model (ΔOFV ≈ 11.7), the additional structural complexity was not supported by sufficiently precise parameter estimation under the predominantly trough-oriented sampling design, particularly for the peripheral volume term (V2, RSE 69%). The one-compartment model was therefore retained as the most parsimonious and clinically interpretable structure. IIV on V was initially evaluated but was not supported by the limited information content of the predominantly late sampling design and was consequently excluded from the final model.

Given the established role of renal function in linezolid elimination, renal function was evaluated a priori as a covariate on CL. Among the renal function descriptors evaluated, absolute eGFR showed the strongest association with CL and was retained in the final model. The effect was incorporated using a centered power function normalized to the population median value of 59.56 mL/min. A progressive decline in apparent linezolid CL over the course of therapy was identified in the dataset. This relationship is illustrated in Supplementary Figure S2, which shows the model-predicted effect of treatment duration on apparent CL with other covariates fixed to their median values. Time-dependent effects were explored using both piecewise and continuous functions, with a continuous power model centered at 3.5 days providing the best balance between statistical improvement and physiological plausibility. Age also demonstrated an independent influence on clearance within the studied range (65–87 years) and was incorporated using a centered power model normalized to 78 years. The typical V was estimated at 25.6 L, consistent with published values in adult populations.

Final model equation for CL (functional form):

$$CLi\;\left(\frac{L}{h}\right)=4.25\times \left({\left(\frac{eGFRi}{59.6}\right)}^{0.29}\right)\times \left({\left(\frac{DAYi}{3.5}\right)}^{-0.18}\right)\times \left({\left(\frac{AGEi}{78}\right)}^{-1.16}\right)\times exp(\eta CL,i)$$

where:

*CLi* is the *CL* of the *i*th individual;

DAYi: treatment duration in days of the ith individual;

*AGEi*: age in years of the *i*th individual;

*eGFRi*: estimated glomerular filtration rate (CKD-EPI formula) in mL/min of the *i*th individual;

*ηCL,i*~N(0, ω^2^CL).

IIV on CL followed a log-normal distribution and decreased from 42.9% in the base structural model to 33.2% in the final covariate model, reflecting improved explanation of between-subject variability after the inclusion of renal function, treatment duration, and age. RUV was best described by a proportional error model. Additive and combined error structures were evaluated but did not improve OFV or diagnostic performance compared with the proportional model.

### 3.3. Model Evaluation and Validation

All fixed-effect parameters were estimated with acceptable precision, with relative standard errors (RSE) below 43% for all structural and covariate parameters. The eta-shrinkage for CL was low (4.3%), indicating robust estimation of individual random effects and adequate information content despite the predominantly late sampling design.

Bootstrap analysis (1000 replicates) confirmed the robustness and stability of the final model. No clinically meaningful differences were observed between the median parameter estimates obtained from the bootstrap procedure and those derived from the original dataset (Table 2). All final parameter estimates were contained within the corresponding 95% confidence intervals obtained from the bootstrap distributions. Of the 1000 bootstrap runs, 10 (1.0%) were excluded due to convergence to boundary estimates, while the remaining 990 successful replicates supported model stability and parameter reliability. Confidence intervals for covariate effects remained consistent across resampled datasets, reinforcing the robustness of the identified predictors.

**Figure 1:**
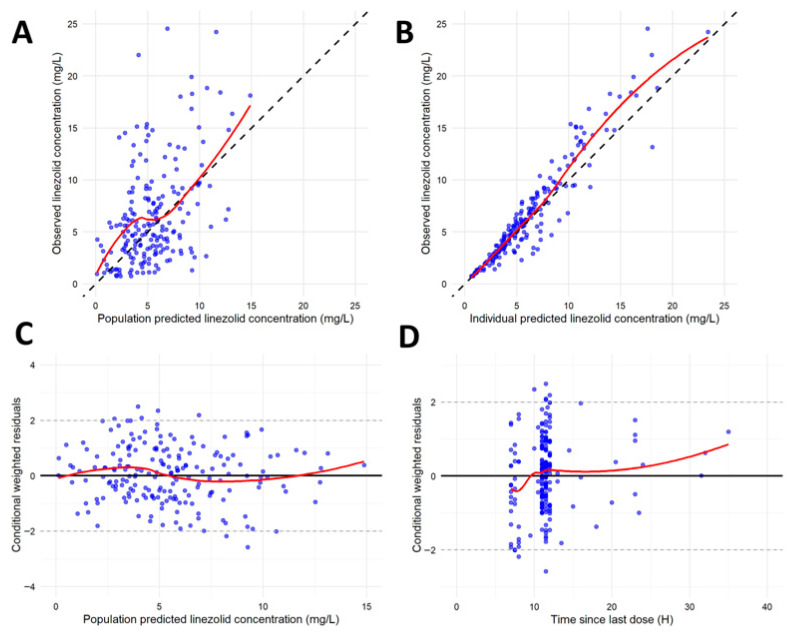
Goodness-of-fit plots for the final population model. (**A**) Observed concentrations versus population-predicted concentrations; (**B**) observed concentrations versus individual-predicted concentrations; (**C**) conditional weighted residuals versus observed concentrations; (**D**) conditional weighted residuals versus time. Black solid line: line of identity; blue circles: linezolid concentrations; red solid line: locally weighted scatterplot smoothing (LOWESS).

The GOF plots (Figure 1) demonstrated adequate agreement between the observed and both population-predicted and individual-predicted concentrations, with no evidence of systematic bias. CWRES were symmetrically distributed around zero without apparent time-dependent trends.

**Figure 2:**
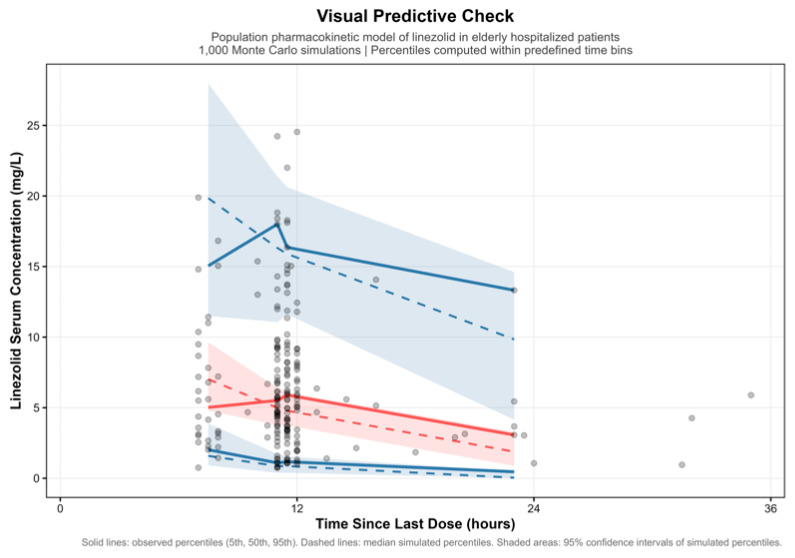
Visual predictive check (VPC) for the concentration–time after-dose profiles of linezolid in elderly patients (internal evaluation). The *x*-axis represents the actual recorded time since the last administered dose for each observation, rather than the nominal dosing interval at treatment initiation. Gray open circles: linezolid observations (Obs); red solid line: median of the Obs; blue solid line: median of the predicted linezolid concentrations (Pred); red dashed lines: 5th and 95th percentiles of the Obs; blue dashed lines, 5th and 95th percentiles of the Pred (90% prediction interval, PI); blue-shaded area: 95% confidence interval (CI) for the 5th, 50th, and 95th percentiles of the Pred; red-shaded area: 95% CI for the 5th, 50th, and 95th percentiles of the Obs. The VPC was based on 1000 Monte Carlo simulations over a 36-h period following the last dose in 198 observations from the development dataset.

A VPC based on 1000 Monte Carlo simulations showed that the model adequately reproduced both the central tendency and dispersion of the observed serum concentration–time since last dose data (Figure 2). The majority of observed concentrations were contained within the 95% prediction intervals, with no evidence of systematic deviation over time. Overall, these results support the ability of the final model to reliably describe and predict linezolid pharmacokinetics in elderly hospitalized patients.

**Figure 3:**
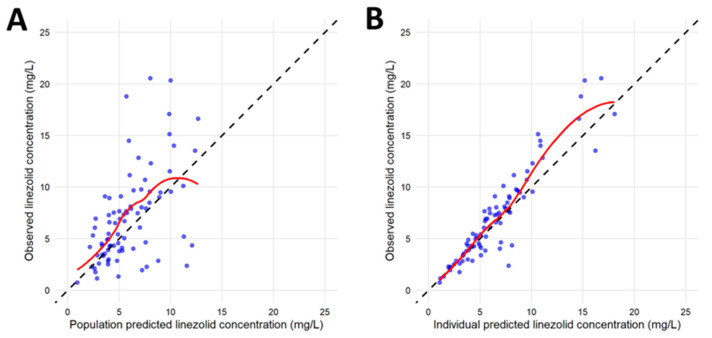
Goodness-of-fit plots for the independent validation cohort. (**A**) Observed concentrations versus population-predicted concentrations; (**B**) observed concentrations versus individual-predicted concentrations. Black dashed line: line of identity; blue circles: observed linezolid concentrations; red solid line: locally weighted scatterplot smoothing (LOWESS).

Independent validation was performed using the cohort reserved for model evaluation. Graphical diagnostics in the validation dataset showed satisfactory predictive performance. GOF plots of observed versus population-predicted concentrations demonstrated acceptable agreement without evidence of systematic bias across the concentration range. Plots of observed versus individual-predicted concentrations showed improved correlation at the individual level, supporting the model’s ability to adequately describe IIV in an independent cohort (Figure 3). Predictive performance was quantified using the MPE and MAPE. In the validation cohort, the final model yielded an MPE of −10.54%, indicating a modest tendency toward overprediction, and a MAPE of 47.8%, reflecting moderate predictive imprecision under real-world conditions with sparse sampling. Overall, these findings support the generalizability of the final model for describing linezolid pharmacokinetics in elderly hospitalized patients.

### 3.4. Stochastic Simulations and Probability of Target Attainment

**Figure 4:**
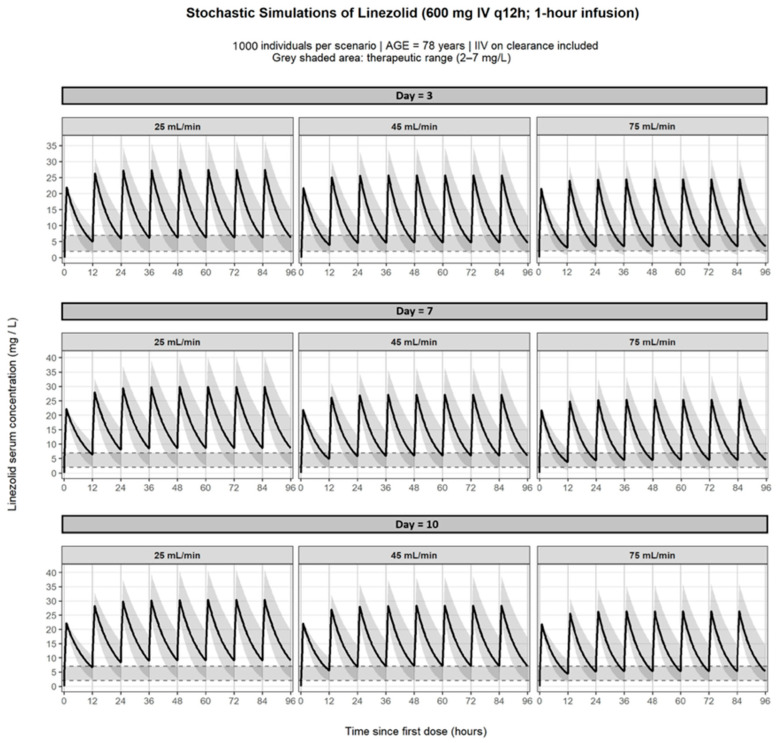
Stochastic simulations of linezolid concentration–time profiles under standard dosing (600 mg IV every 12 h; 1-h infusion) across representative renal function and treatment-duration scenarios. Nine panels are shown, corresponding to three treatment days (Day 3, Day 7, and Day 10) and three absolute estimated glomerular filtration rate (eGFRabs) values (25, 45, and 75 mL/min). Solid lines represent the median simulated concentrations, and shaded areas represent the 95% confidence intervals derived from 1000 virtual individuals per scenario, with interindividual variability on clearance retained. Age was fixed at 78 years. The grey shaded horizontal band indicates the therapeutic trough concentration range (2–7 mg/L), and the dashed horizontal lines indicate its lower and upper limits.

Monte Carlo simulations were performed using the final PopPK model to characterize linezolid exposure under standard dosing (600 mg IV every 12 h; 1-h infusion) across clinically relevant renal function and treatment-duration scenarios (Figure 4). Renal function was defined according to the 25th, 50th, and 75th percentiles of the study population (absolute eGFR 25, 45, and 75 mL/min, respectively), ensuring representativeness of the elderly cohort. Treatment duration was explored at 3, 7, and 10 days, reflecting early, intermediate, and prolonged therapy within the observed treatment range. Age was fixed at 78 years (population median).

Simulated concentration–time profiles showed that treatment durations beyond 7 days were associated with a progressive increase in linezolid exposure, particularly in patients with reduced renal function. At day 3, patients with severe renal impairment (eGFR 25 mL/min) already exhibited a high probability of Cmin exceeding the upper therapeutic threshold (7 mg/L), whereas individuals with preserved renal function (75 mL/min) largely remained within the therapeutic range. By day 7, accumulation became more pronounced; patients with eGFR 25 mL/min showed sustained concentrations above 7 mg/L throughout the dosing interval, and notably, even patients with moderate renal impairment (45 mL/min) displayed an increased likelihood of exceeding the upper therapeutic limit. At day 10, the risk of overexposure further increased, with both the severe (25 mL/min) and moderate (45 mL/min) renal function groups demonstrating a substantial probability of concentrations above 7 mg/L, while patients with preserved renal function maintained comparatively lower exposure levels.

**Figure 5:**
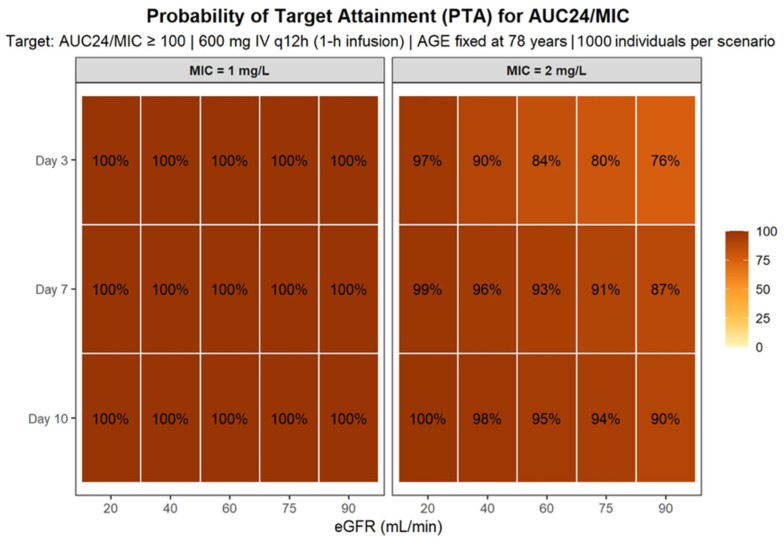
Probability of target attainment (PTA) heatmap for the pharmacodynamic index AUC24/MIC under standard linezolid dosing. Tiles represent the predicted probability (percentage) of achieving the predefined target (AUC24/MIC ≥ 100) for each combination of absolute eGFR (eGFR) and treatment day (Day 3, Day 7, and Day 10), stratified by MIC (1 and 2 mg/L). Simulations were performed using the final population pharmacokinetic model assuming linezolid 600 mg IV every 12 h (1-h infusion), with age fixed at 78 years and interindividual variability on clearance retained (1000 individuals per scenario). Individual AUC24 values were obtained for each virtual subject by numerical integration of the model-predicted concentration–time profile over each 24-h interval on a dense simulation grid, and PTA was calculated as the proportion of individuals meeting the AUC24/MIC target for each MIC scenario.

**Figure 6:**
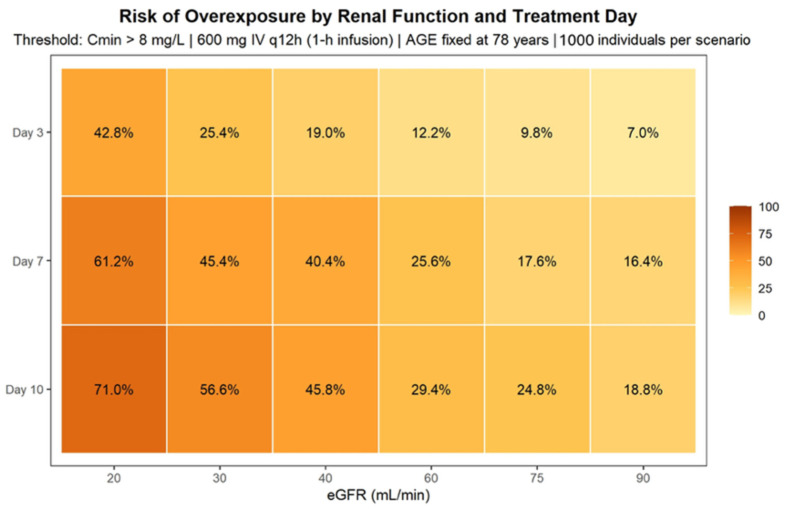
Heatmap of the predicted risk of linezolid overexposure under standard dosing. Tiles represent the percentage of simulated individuals with trough concentrations exceeding 8 mg/L (Cmin > 8 mg/L) for each combination of absolute estimated glomerular filtration rate (eGFRabs) and treatment day (Day 3, Day 7, and Day 10). Simulations were performed using the final population pharmacokinetic model assuming linezolid 600 mg IV every 12 h (1-h infusion), with age fixed at 78 years and interindividual variability on clearance retained (1000 individuals per scenario). Values are shown for representative renal function scenarios (eGFRabs 20, 40, 60, 75, and 90 mL/min).

The probability of achieving potentially toxic Cmin (>8 mg/L) increased markedly with both declining renal function and longer treatment duration. At day 3, this probability was already clinically relevant in patients with eGFR 25 mL/min. By days 7 and 10, the risk substantially increased in both the 25 and 45 mL/min scenarios, whereas it remained low in patients with eGFR 75 mL/min. These findings suggest that under standard dosing, elderly patients with moderate-to-severe renal impairment are at considerable risk of supratherapeutic exposure during prolonged therapy. The PTA was further evaluated using the AUC24/MIC index for MIC values of 1 and 2 mg/L. For MIC = 1 mg/L, standard dosing achieved high PTA across most renal function scenarios, particularly beyond day 3. In contrast, for MIC = 2 mg/L, PTA was reduced in patients with preserved renal function (75 mL/min), especially at earlier time points, whereas patients with moderate and severe renal impairment maintained higher probabilities of target attainment due to increased systemic exposure (Figure 5). These simulations illustrate the exposure–toxicity trade-off inherent to linezolid therapy in elderly patients, whereby renal impairment enhances pharmacodynamic target attainment but simultaneously increases the risk of concentration-dependent toxicity. Based on these results, model-informed nomograms were developed to estimate the probability of toxic exposure (>8 mg/L) and to support individualized dosing strategies according to renal function and treatment duration (Figure 6).

## 4. Discussion

Linezolid has a narrow therapeutic window, with efficacy driven by the AUC/MIC ratio and toxicity associated with elevated Cmin. Despite this, a fixed dose of 600 mg every 12 h is commonly administered irrespective of patient characteristics [1,2,3,4,5]. In elderly patients, age-related physiological changes and comorbidities may alter linezolid pharmacokinetics, increasing the risk of hematological toxicity, particularly thrombocytopenia [15,16]. However, no specific dosing recommendations currently exist for this age group, highlighting the need for PopPK models to support TDM-guided dose individualization.

In this study, linezolid pharmacokinetics were adequately described by a one-compartment model with first-order elimination, consistent with previous models developed under sparse TDM sampling. As noted by Bandín-Vilar et al., limited sampling per patient often precludes the reliable estimation of additional parameters required for two-compartment models [27]. Age-related changes in body composition may reduce distributional complexity in elderly patients, supporting the adequacy of a parsimonious structural model [28]. A two-compartment model did not meaningfully improve model fit and showed poor parameter precision. Renal function, age, and treatment duration were identified as significant covariates influencing CL. The robustness of the model was supported by bootstrap analysis and by the very low shrinkage observed for clearance (4.3%), indicating that individual data were highly informative and that model-based individual predictions are reliable.

The estimated CL (4.25 L/h) and V (25.6 L) were lower than those typically reported in broader adult populations, where higher values are commonly observed [27]. These findings likely reflect the advanced age and greater prevalence of renal impairment in our cohort. Notably, our CL estimate closely aligns with that reported by Li et al. in hospitalized elderly patients, suggesting that values around 4.2–4.3 L/h may represent a consistent pharmacokinetic benchmark in older inpatient populations [29]. In contrast, higher CL values described in studies including younger individuals are consistent with wider age ranges and better renal function [7]. Similarly, the reduced V observed in our study is in agreement with the 27.6 L reported by Matsumoto et al. in elderly Japanese patients with low body weight and renal impairment [30], supporting the concept that contracted distribution volumes are a characteristic pharmacokinetic feature of this population, likely related to age-associated reductions in lean body mass and total body water. The present study adds to previous linezolid PopPK reports by focusing on elderly hospitalized patients in routine clinical practice and by highlighting the combined influence of renal function, age, and treatment duration on drug exposure.

Renal function was the most robust covariate identified in the model, showing a clear positive association with linezolid CL. This finding is consistent with PopPK studies that have consistently identified renal function as the main determinant of variability in linezolid elimination [30,31]. Beyond its statistical relevance, the clinical impact of renal impairment is well-established, with higher SLC and an increased risk of hematological toxicity reported in patients with reduced eGFR [16,31]. In our elderly cohort, where renal dysfunction was common and often dynamic during hospitalization, these findings further support the need for renal function-guided dosing and early TDM.

Age was identified as an independent negative predictor of CL, indicating that even within this exclusively elderly cohort, older patients exhibited lower linezolid elimination than those in their mid-sixties. Formal collinearity assessment showed only a weak association between age and renal function (Spearman’s ρ = −0.203) with a low variance inflation factor (VIF = 1.05), suggesting limited collinearity between these covariates. Therefore, despite the inclusion of age in the CKD-EPI equation, AGE appeared to provide additional explanatory information beyond eGFR in this dataset. This effect remained statistically robust and consistent with previous reports describing age-related reductions in clearance [7,31]. While some models developed in homogeneous elderly populations have not retained age as a significant covariate, clinical evidence suggests that aging per se contributes to linezolid accumulation independently of renal decline [8,10]. These findings support the concept that mechanisms beyond renal function, including age-related physiological changes and altered body composition, may further impair drug elimination in the oldest patients.

A progressive decline in apparent linezolid CL over the course of treatment was identified as a significant covariate in the model. This finding should be interpreted cautiously, as in a retrospective real-world dataset, it may reflect not only time-dependent pharmacokinetic changes but also concurrent changes in clinical status, inflammation, renal function, dose adaptation after TDM, or survivor bias. Therefore, this covariate should be regarded primarily as a phenomenological description of the observed data rather than as direct evidence of a specific biological mechanism. Nevertheless, its inclusion improved model fit and may still be clinically useful to support repeated TDM during prolonged therapy.

To our knowledge, this time-dependent behavior has not been formally quantified in a population pharmacokinetic model developed exclusively in elderly patients and represents a novel finding of the present study. Although the underlying mechanism remains incompletely understood, previous studies have described time-dependent or nonlinear CL of linezolid, suggesting progressive inhibition of elimination pathways during prolonged exposure [32,33,34]. Clinically, this finding provides a pharmacokinetic explanation for the well-recognized temporal pattern of linezolid toxicity, particularly thrombocytopenia, which is strongly associated with treatment durations beyond 10–14 days [5,16]. These results support the need for repeated TDM rather than reliance on a single early measurement.

The inclusion of renal function, age, and treatment duration reduced the IIV in CL from 42.9% to 33.2% CV, indicating that these covariates explain a substantial proportion of between-subject variability in this elderly cohort. This residual variability, although lower than that reported in broader adult populations [7,31], likely reflects unmeasured factors common in hospitalized older patients, such as comorbidities, polypharmacy, and dynamic clinical instability. RUV remained moderate (26.5% CV), consistent with real-world clinical TDM data and attributable to expected sources such as sampling imprecision and analytical variability. Overall, these findings reinforce the need for Bayesian TDM to achieve reliable individual dose optimization.

The predictive performance of the final model was evaluated in a randomly allocated validation cohort. The model showed low bias (MPE −10.5%) but only moderate precision (MAPE 47.8%), indicating limited a priori predictive performance. This should be interpreted in the context of published external evaluations of linezolid PopPK models. Qin et al. reported that none of the 25 published models met the predefined acceptability criteria in external prediction-based diagnostics, with median MAPE values ranging from 58.25% to 72.59% depending on the population assessed. Predictive performance improved substantially after Bayesian updating with prior observations. Taken together, these data suggest that linezolid PopPK models, including ours, are more appropriately used as support tools for Bayesian TDM than as standalone a priori predictors of individual exposure [35]. In this context, the present predictive performance aligns with expectations for population-based predictions in hospitalized elderly patients. Although derived from the same institution, the inclusion of an independent validation cohort strengthens the credibility of the model, as formal external validation remains uncommon in published linezolid PopPK studies [27,36].

Stochastic simulations under standard dosing (600 mg IV every 12 h) demonstrated a clear and progressive increase in linezolid exposure with declining renal function and advancing treatment duration. At Day 3, patients with preserved renal function (75 mL/min) predominantly remained within the therapeutic range (2–7 mg/L), whereas those with severe renal impairment (25 mL/min) already exhibited Cmin approaching or exceeding the upper safety threshold. By Day 7, accumulation became evident across renal strata, and by Day 10, patients with moderate renal dysfunction (45 mL/min) frequently displayed sustained Cmin > 7 mg/L, indicating clinically relevant overexposure. The widening prediction intervals over time further reflect the combined impact of IIV and time-dependent CL reduction, resulting in progressively greater dispersion of exposure trajectories. Probability-based analyses confirmed this exposure–toxicity pattern. The risk of supratherapeutic Cmin (>8 mg/L) increased markedly with both declining eGFR and longer treatment duration, reaching approximately 70% at Day 10 in patients with eGFR 20–25 mL/min and approaching 45–50% in those with moderate renal impairment. In contrast, pharmacodynamic target attainment (AUC24/MIC ≥ 100) was uniformly achieved for MIC = 1 mg/L across all renal strata and treatment days. For MIC = 2 mg/L, target attainment remained high even in patients with preserved renal function and approached near-universal attainment in those with renal impairment. Together, these findings highlight a clinically relevant exposure–toxicity imbalance in elderly hospitalized patients: while efficacy targets are consistently achieved under standard dosing, the probability of concentration-dependent toxicity increases substantially with renal dysfunction and prolonged therapy. These results suggest that beyond the first treatment week and particularly in patients with eGFR below approximately 45 mL/min, dose adjustment strategies should primarily aim to mitigate cumulative overexposure rather than to enhance pharmacodynamic efficacy. Overall, these findings indicate that in elderly hospitalized patients, standard dosing reliably achieves efficacy targets but is associated with an increasing risk of toxicity, particularly beyond the first treatment week and in patients with reduced renal function. From a clinical perspective, these findings support proactive TDM in elderly patients receiving linezolid, particularly in those with reduced renal function (eGFR < 45 mL/min), who may benefit from early dose reassessment and monitoring. Although formal simulations of alternative starting regimens were beyond the scope of the present study, the observed risk patterns suggest that an initial dose reduction strategy could be considered in selected high-risk patients, provided that it is promptly supported by therapeutic drug monitoring.

This study presents several strengths. It was conducted in a well-characterized cohort of elderly hospitalized patients (65–87 years) with a broad spectrum of renal function, reflecting routine clinical practice. Renal function was modeled using absolute eGFR, enhancing physiological coherence with drug CL. The identification of treatment duration as a time-dependent determinant of CL provides clinically relevant insight for prolonged therapy in this population. The study also incorporates a randomly allocated validation cohort, an approach infrequently implemented in real-world PopPK studies. Model robustness was supported by stable bootstrap results and low shrinkage for CL, indicating reliable individual predictions. Finally, the translation of model findings into probability-based simulations focused on toxicity risk enhances the clinical applicability of this work.

The predominantly trough-oriented late sampling design limited the characterization of distribution kinetics, precluded a reliable estimation of IIV on V, and may have favored the selection of a one-compartment structure. Thus, despite acceptable precision and bootstrap stability for the typical V estimate, the model should be interpreted as a pragmatic TDM-oriented tool rather than a fully descriptive structural model, with some uncertainty remaining about the full PK profile and model-based AUC estimates. Linezolid concentrations were measured using an immunoassay rather than LC-MS/MS, which may have contributed to residual variability. Although key covariates were incorporated, residual confounding from unmeasured clinical factors common in elderly hospitalized patients, such as frailty, polypharmacy, inflammation, or dynamic changes in organ function, cannot be excluded; in this context, the time-dependent CL effect may partially reflect evolving clinical status rather than a purely pharmacokinetic phenomenon. Although derived from the same institution and study period, inclusion of a randomly allocated independent validation cohort strengthens internal model evaluation; however, formal external validation in an independent population is still needed. Finally, the single-center design and exclusion of critically ill ICU patients may limit generalizability to other healthcare settings or more severely ill populations.

## 5. Conclusions

This study presents a validated PopPK model of linezolid developed exclusively in elderly hospitalized patients, identifying renal function, age, and treatment duration as independent determinants of CL variability. The observed association between treatment duration and reduced apparent CL may contribute to cumulative overexposure during prolonged therapy, particularly in patients with impaired renal function. These findings highlight the clinical importance of pharmacokinetic variability in elderly inpatients and support the use of the model for safety-oriented Bayesian dose individualization, as well as early and repeated therapeutic drug monitoring in this vulnerable population.

## Figures and Tables

**Table 1:** Demographic and clinical data of patients in the development and validation cohorts.

Characteristics	Development Cohort	Validation Cohort
Total number of patients	103	46
Sex (male), n (%)	67 (65.0)	24 (52.2)
Age, median [range], years	78 [65–87]	79.5 [65–86]
Weight, median [IQR], kg	70 [60–80]	66.5 [59.3–75.9]
Ideal body weight, median [IQR], kg	58.5 [54–64.3]	58.8 [54.4–63.5]
Body mass index (BMI), median [IQR], kg/m^2^	26.6 [23.8–30.0]	24.2 [22.1–28.1]
Underweight (BMI < 18.5 kg/m^2^), n (%)	1 (1.0)	1 (2.2)
Normal weight (BMI 18.5–24.9 kg/m^2^), n (%)	39 (37.9)	25 (54.3)
Overweight (BMI 25.0–29.9 kg/m^2^), n (%)	39 (37.9)	12 (26.1)
Obese (BMI > 30 kg/m^2^), n (%)	24 (23.3)	8 (17.4)
**Renal function**		
Estimated glomerular filtration rate, median [IQR], mL/min	48.8 [25.9–75.4]	40.3 [24.1–61.4]
>90 mL/min	12 (11.7)	3 (6.5)
60–89 mL/min	27 (26.2)	9 (19.6)
30–59 mL/min	33 (32.0)	17 (37.0)
<30 mL/min	31 (30.1)	17 (37.0)
**Diagnosis**		
Respiratory infection	22 (21.4)	11 (23.9)
Skin and soft tissue infection	23 (22.3)	4 (8.7)
Urinary tract infection (UTI)	24 (23.3)	9 (19.6)
Other infections	34 (33.0)	22 (47.8)
**Biochemical parameters, median [IQR]**		
Alanine aminotransferase (ALT), U/L	24 [11.5–36.5]	18 [12.5–31.0]
Aspartate aminotransferase AST, U/L	44 [30–77]	45.5 [37.5–62.2]
Total bilirubin (BLT), mg/dL	0.4 [0.3–0.6]	0.4 [0.3–0.6]
Lactate dehydrogenase (LDH), U/L	212.0 [174.5–285.5]	253 [191.0–314.5]
Total protein, g/dL	5.5 [5.1–6.0]	5.5 [5.2–5.9]
Albumin, g/dL	3.0 [2.7–3.3]	3.1 [2.9–3.3]
C-Reactive protein, mg/L	9.4 [4.0–19.2]	11.3 [6.6–16.1]
Procalcitonin, ng/mL	0.5 [0.2–1.3]	0.3 [0.2–0.6]
Hemoglobin, g/dL	10.1 [9.0–11.6]	10.3 [9.2–11.9]
Platelets, ×10^9^/L	241 [172.5–349.5]	255 [189.8–354.3]
**Treatment**		
Duration of linezolid treatment, median [range], days	7 [3–26]	6 [3–18]
Daily dose per body weight [IQR], mg/kg/day	14.7 [10.0–17.7]	15.3 [10–19.35]
Daily dose, median [range], mg/day	1200 [300–1800]	1200 [300–1800]
Maximum daily dose, median [range], mg/day	1200 [600–2400]	1200 [600–2400]
**Concomitant medication, n (%)**		
Proton pump inhibitors	88 (85.4)	42 (91.3)
Azole antifungals	13 (12.6)	8 (17.4)
Levothyroxine	6 (5.8)	7 (15.2)
Amiodarone	1 (1.0)	1 (2.2)
Macrolides	7 (6.8)	4 (8.7)
Rifampicin	3 (2.9)	0 (0)
**Linezolid drug monitoring**		
Plasma samples, n	198	95
TDM/patient, median [range]	2 [1–5]	2 [1–6]
Days until first TDM measurement, median [IQR], days	3 [2–4]	3 [2–4]
Mean concentration (SD), mg/L	6.5 (4.0)	7.5 (4.3)

**Table 2:** Linezolid population pharmacokinetic parameters.

Parameters	Final Model Estimate	RSE (%)	Shrinkage	Bootstrap (n = 1000)	
				Mean	95% CI
CLpop (L/h)	4.25	9	4.3	4.22	3.76–4.82
eGFR-CL	0.29	16		0.28	0.18–0.38
DAY-CL	−0.18	14		−0.18	−0.25–−0.12
AGE-CL	−1.16	43		−1.17	−1.90–−0.46
Vpop (L)	25.60	17		25.40	21.8–30.35
IIVCL (CV, %)	33.20	9		33.20	28.3–36.10
RUVprop (CV, %)	26.50	17		26.50	24.50–30.00

AGE, age in years; AGE-CL, magnitude of the effect of AGE on CL; CI, confidence interval; CLpop, clearance of the typical subject; CV, coefficient of variation; DAY, treatment duration in days; DAY-CL, magnitude of the effect of DAY on CL; eGFR, estimated glomerular filtration rate (CKD-EPI formula) in mL/min; eGFR-CL, magnitude of the effect of eGFR on CL; IIVCL, interindividual variability on clearance; RSE, relative standard error; RUVprop, proportional residual unexplained variability; Vpop, typical population value of volume of distribution.

## Data Availability

The original contributions presented in this study are included in the article. Further inquiries can be directed to the corresponding author.

## Supplementary Materials

The following supporting information can be downloaded at: https://www.mdpi.com/article/10.3390/pharmaceutics18050528/s1, Figure S1: Observed linezolid concentrations plotted against time since the first administered dose, Figure S2: Model-predicted effect of treatment duration on apparent linezolid clearance.

## Abbreviations

The following abbreviations are used in this manuscript:

AUC/MIC	Ratio between the area under the concentration–time curve and the minimum inhibitory concentration
BMI	Body mass index
BSA	Body surface area
CL	Drug clearance
Cmin	Trough concentration (immediately before the next dose)
CWRES	Conditional weighted residuals
eGFR	Estimated glomerular filtration rate
IBW	Ideal body weight
MAPE	Mean absolute prediction error
MPE	Mean prediction error
MRSA	Methicillin-resistant *Staphylococcus aureus*
OFV	Objective function value
PopPK	Population pharmacokinetic
PTA	Probability of target attainment
RSE	Relative standard error
RUV	Residual unexplained variability
SLC	Serum linezolid concentration
TDM	Therapeutic drug monitoring
V	Volume of distribution
VPC	Visual predictive check
VRE	Vancomycin-resistant *Enterococcus faecium*
