# Supplementary material for: Population Pharmacokinetics of Linezolid in Elderly Hospitalized Patients: Implications for Therapeutic Drug Monitoring
Source: Pharmaceutics. 2026 Apr 27;18(5):528. doi: 10.3390/pharmaceutics18050528 (PMC13210461; doi:10.3390/pharmaceutics18050528)
Supplement: Supplementary file 1 [file pharmaceutics-18-00528-s001.zip › pharmaceutics-4233303-supplementary.pdf]

Supplementary Figure S1. Observed linezolid concentrations versus time since the first dose.

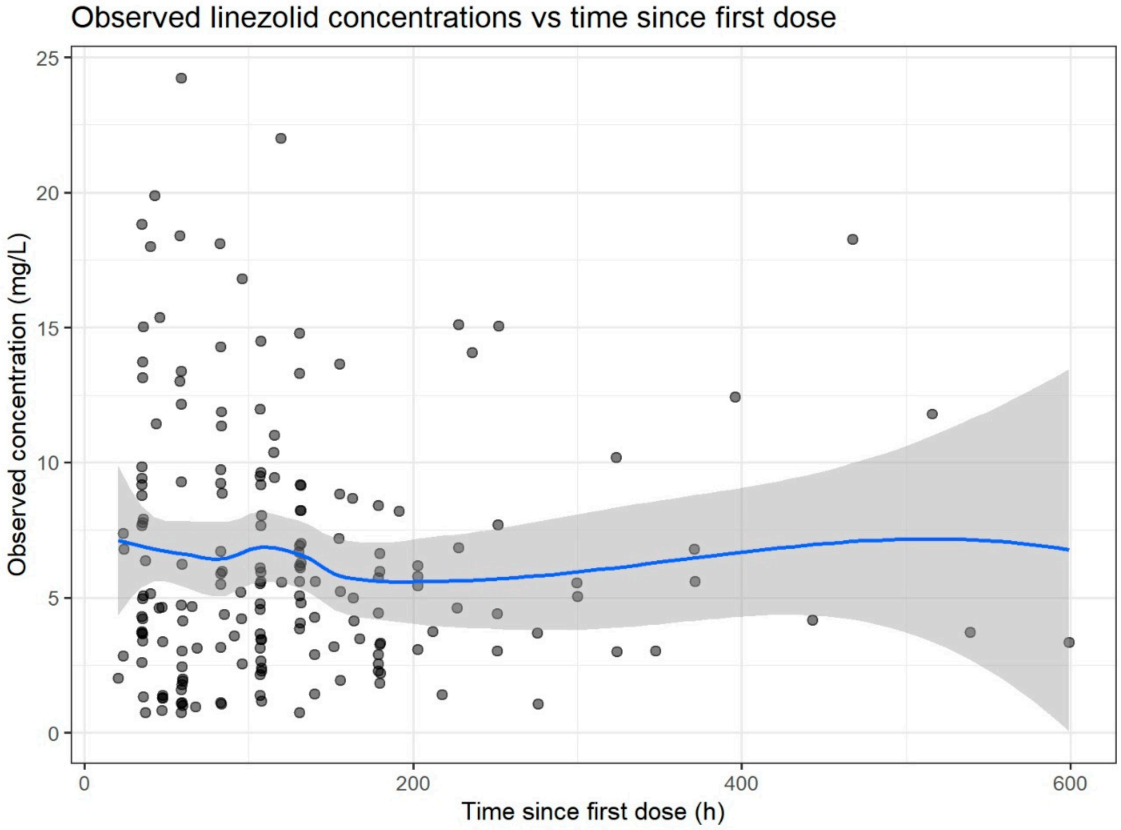

Supplementary Figure S1. Observed linezolid concentrations plotted against time since the first administered dose. Points represent individual observed serum concentrations. The solid line shows a locally weighted smoothing (LOESS) curve, and the shaded area represents its 95% confidence interval. The figure illustrates the temporal distribution of the raw concentration data and the predominantly trough-oriented sampling obtained during routine therapeutic drug monitoring.

Supplementary Figure S2. Model-predicted effect of treatment duration on apparent linezolid clearance.

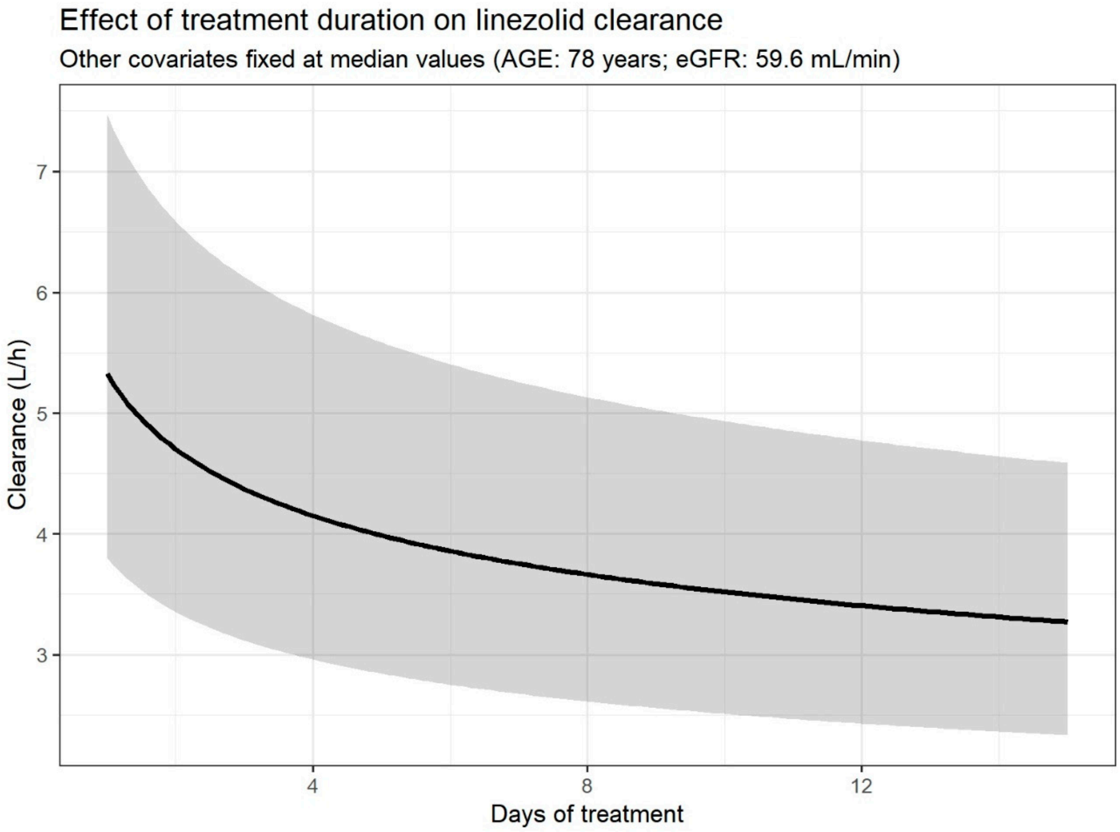

Supplementary Figure S2. Model-predicted effect of treatment duration on apparent linezolid clearance. The solid line represents the typical apparent clearance predicted by the final population pharmacokinetic model across treatment duration, with age and eGFR fixed to their median values (78 years and 59.56 mL/min, respectively). The shaded area reflects the variability around the predicted clearance profile according to the estimated interindividual variability in the model.
